# Synergistic interaction between APOE and family history of Alzheimer’s disease on cerebral amyloid deposition and glucose metabolism

**DOI:** 10.1186/s13195-018-0411-x

**Published:** 2018-08-23

**Authors:** Dahyun Yi, Younghwa Lee, Min Soo Byun, Jun Ho Lee, Kang Ko, Bo Kyung Sohn, Young Min Choe, Hyo Jung Choi, Hyewon Baek, Chul-Ho Sohn, Yu Kyeong Kim, Dong Young Lee, for the KBASE research group

**Affiliations:** 1 Institute of Human Behavioral Medicine, Medical Research Center Seoul National University, Seoul, Republic of Korea; 2 Department of Neuropsychiatry, Seoul National University Hospital, Seoul, Republic of Korea; 3 Department of Psychiatry, Sanggye Paik Hospital, Inje University College of Medicine, Seoul, Republic of Korea; 4 Department of Neuropsychiatry, University of Ulsan College of Medicine, Ulsan University Hospital, Ulsan, Republic of Korea; 5 Department of Neuropsychiatry, SMG-SNU Boramae Medical Center, Seoul, Republic of Korea; 6 Department of Neuropsychiatry, Kyunggi Provincial Hospital for the Elderly, Yongin, Republic of Korea; 7 Department of Radiology, Seoul National University College of Medicine & Seoul National University Hospital, Seoul, Republic of Korea; 8 Department of Nuclear Medicine, SMG-SNU Boramae Medical Center, Seoul, Republic of Korea; 9 Department of Psychiatry, Seoul National University College of Medicine, 101 Daehak-ro, Jongno-gu, Seoul, 03080 Republic of Korea

**Keywords:** APOE, Family history of Alzheimer’s disease, Cognitively normal adults, Amyloid beta deposition, Cerebral glucose metabolism

## Abstract

**Background:**

Recently, the field of gene-gene or gene-environment interaction research appears to have gained growing interest, although it is seldom investigated in Alzheimer’s disease (AD). Hence, the current study aims to investigate interaction effects of the key genetic and environmental risks—the apolipoprotein ε4 allele (APOE4) and family history of late-onset AD (FH)—on AD-related brain changes in cognitively normal (CN) middle-aged and older adults.

**Methods:**

[^11^C] Pittsburg compound-B (PiB) positron emission tomography (PET) imaging as well as [^18^F] fluoro-2-deoxyglucose (FDG) PET that were simultaneously taken with T1-weighted magnetic resonance imaging (MRI) were obtained from 268 CNs from the Korean Brain Aging Study for Early Diagnosis and Prediction of AD (KBASE). Composite standardized uptake value ratios were obtained from PiB-PET and FDG-PET images in the AD signature regions of interests (ROIs) and analyzed. Voxel-wise analyses were also performed to examine detailed regional changes not captured by the ROI analyses.

**Results:**

A significant synergistic interaction effect was found between the APOE4 and FH on amyloid-beta (Aβ) deposition in the AD signature ROIs as well as other regions. Synergistic interaction effects on cerebral glucose metabolism were observed in the regions not captured by the AD signature ROIs, particularly in the medial temporal regions.

**Conclusions:**

Strong synergistic effects of APOE4 and FH on Aβ deposition and cerebral glucose metabolism in CN adults indicate possible gene-to-gene or gene-to-environment interactions that are crucial for pathogenesis of AD involving Aβ. Other unspecified risk factors—genes and/or environmental—that are captured by the positive FH status might either coexpress or interact with APOE4 to alter AD-related brain changes in CN. Healthy people with both FH and APOE4 need more attention for AD prevention.

**Electronic supplementary material:**

The online version of this article (10.1186/s13195-018-0411-x) contains supplementary material, which is available to authorized users.

## Background

Decades of research on sporadic late-onset Alzheimer’s disease (AD) dementia, the most common form of dementia, have shown that AD dementia is a multifactorial disease with a wide variety of genetic and environmental factors playing a role in the age of onset, risk, and etiology [1]. Numerous heritable and inheritable risk factors have been linked with AD pathogenesis. Nevertheless, to date, only a limited number of studies have investigated the interaction effect between the major risks on in-vivo AD-related brain changes which can reflect the pathogenesis of AD. Information about whether the major risks of AD dementia can synergistically increase AD-specific brain changes before the onset of dementia symptoms will likely help identification of a more urgent target population for the preventive efforts against AD dementia.

The apolipoprotein ε4 allele (APOE4) is a major risk factor for AD dementia and is associated with a decade or more mean age at onset decrease in AD symptoms [2]. Numerous studies have reported a relationship between APOE4 and AD-related brain changes such as increased amyloid-beta (Aβ) deposition and decreased glucose metabolism even in cognitively normal (CN) elderly individuals, although with some inconsistencies in the degree of effects [3–9]. Notably, functional brain abnormalities associated with APOE4 as identified by decreased cerebral glucose metabolism in the AD-related brain regions were found in healthy volunteers even as young as 20–39 years old [10]. Furthermore, a recent meta-analysis has shown strong effects of APOE4 on not only the prevalence of amyloid pathology but also the age of onset of AD dementia [11]. Despite some inconsistencies, the presence of strong effects of APOE4 on Aβ deposition and cerebral glucose metabolism has been repeatedly and undeniably shown.

In recent years, evidence has accumulated for other genetic and environmental factors that influence AD-related brain changes [1]. Having a first-degree family history of AD dementia (FH) is another well-known risk factor for developing AD dementia and is considered to encapsulate both genetic and environmental risk loads [12] as it not only captures heritable genetic susceptibility but also other shared dietary, psychosocial, and somatic factors that are shown to be associated with an individual’s risk for developing AD dementia [13–15]. Similar to APOE4, the effects of FH on AD-related brain changes including Aβ deposition and glucose metabolism are present even in CN individuals, although less consistently [16–18].

Previous research shows that FH and APOE4 highly co-occur [19, 20] and, conceivably, their effects on developing AD dementia may overlap. However, given that they reflect different hereditary factors (i.e., genetic only or both genetic and environmental), it is likely that an interaction of the effects of FH and APOE4 exists for AD-related brain changes such that having both FH and APOE4 will lead to synergistic influences on AD-related brain changes compared to when an individual has only one of the two risk factors. Nonetheless, to the best of our knowledge, no study has examined the synergistic interaction effects of FH and APOE4 on AD-related brain changes, specifically Aβ deposition and glucose metabolism, in cognitively intact adults. In this context, the purpose of the current study was to test the hypothesis that APOE4 and FH have synergistic interaction effects on cerebral Aβ deposition and glucose metabolism in healthy middle-aged and older adults.

## Methods

### Subjects

This study is a part of the Korean Brain Aging Study for the Early Diagnosis and Prediction of AD (KBASE), an ongoing prospective cohort study that began in 2014 designed to identify novel biomarkers for AD and to explore various lifetime experiences contributing to AD-related brain changes. The current study included 268 community-dwelling CN individuals, between 50 years and 87 years of age, who were recruited as of March 2016. Details on the KBASE study characteristics including recruitment have been described previously [21]. Individuals with medical, psychiatric, and/or neurological conditions or history of conditions that may affect brain structures or functions, such as stroke, head trauma, depression, hydrocephalus, or focal brain lesions on magnetic resonance imaging (MRI) were excluded. All subjects had reliable informants available who provided corroborative information on the family history of medical conditions, including the presence of AD dementia. Subjects had a Clinical Dementia Rating of 0 and performed within the normal range relative to age-, gender-, and education-adjusted normative means on comprehensive neuropsychological assessments [22, 23]. The study was ethically reviewed and all participants provided written informed consent to participate in this study after receiving a complete description of the study, which is approved by Seoul National University Hospital Institutional Review Board.

### Assessments

Comprehensive clinical and neuropsychological assessment data were obtained from all participants based on the KBASE assessment protocol that incorporated and expanded upon the Korean version of the Consortium to Establish a Registry for Alzheimer’s Disease assessment packet (CERAD-K) [22, 24]. A detailed description of the cognitive assessments has been previously reported [21]. Briefly, the assessments included the Mini-Mental State Examination in the Korean version of the CERAD assessment packet (MMSE-KC), the CERAD-K verbal memory tests, including Word List Memory, Word List Delayed Recall, Word List Recognition, CERAD-K Constructional Praxis, and CERAD-K nonverbal memory delayed recall, the Trail Making Test A and B, and the Stroop Test (Korean Golden version), Verbal Fluency Tasks (both semantic and phonemic), the CERAD-K confrontational naming test (Modified Korean version of the Boston Naming Test), and the Wechsler Adult Intelligence Scale-revised edition Korean version (WAIS-R-K) Digit Span (forward and backward). Neuropsychological test performances are presented as *z* scores based on age-, gender-, and education-adjusted normative data [24].

Genomic DNA was extracted from whole blood and APOE genotyping was performed [25]; subjects with at least one ε4 allele were identified as APOE4 carriers. For the majority of participants, cognitive assessments were administered on the same day that the neuroimaging scans were conducted; four individuals underwent cognitive assessment and neuroimaging scans on different dates where the interval was less than 1 month.

### Family history of AD

Subjects and reliable informants were administered a semistructured interview by trained psychiatrists or a registered nurse to gather detailed information of any family history of dementia. Participants were asked: 1) if any of their birth parents, natural grandparents, siblings sharing parents, or other relatives had dementia and/or other type of neurological diseases; 2) if so, what was the diagnosis of the affected relative and the age of onset; 3) whether or not the affected family member is deceased; and 4), if deceased, what was the age at death.

Positivity of FH was determined if at least one first-degree relative (parent or sibling) had AD onset at 65 years of age or older and whose diagnosis had been made by a certified clinician. If formal diagnosis for a parent was unavailable due to their old age or age at death that preceded implementation of the established criteria in hospital, participants were asked additional questions to determine if their parent exhibited the symptoms of AD dementia such as cognitive and functional decline consistent with the criteria in the absence of other known causes that could preclude an AD diagnosis; if sufficient information was gathered and findings were deemed consistent with a diagnosis of AD, these subjects were also identified as FH-positive (FH^+^). If none of the first-degree relatives was identified as having AD dementia, subjects were classified as FH-negative (FH^–^).

### Amyloid-beta imaging

All subjects underwent three-dimensional [^11^C] Pittsburg compound B (PiB) positron emission tomography (PET) imaging simultaneously taken with T1-weighted MRI at 3.0 T using a Biograph mMR (PET-MR) scanner (Siemens, Washington DC, USA). Preprocessing steps were performed using Statistical Parametric Mapping 12 (SPM12) (see Additional file 1: Methods for more detail).

Spatial normalization processes were performed on PiB-PET data using Statistical Parametric Mapping 12 (http://www.fil.ion.ucl.ac.uk/spm/software/spm12/) (SPM12) implemented in Matlab 2014a (Mathworks, MA). Static PiB-PET images were coregistered to individual T1 structural images, and transformation parameters for spatial normalization of individual T1 images to a standard MNI (Montreal Neurological Institute) template were calculated, which were then used to spatially normalize the PET images to the MNI template. The spatially normalized PiB-PET images were smoothed with an 8-mm Gaussian filter.

Additional processes were run for PiB-PET data to obtain improved spatial normalization of cerebellar gray matter, which is used as the reference region for intensity normalization (see Additional file 1: Methods section of the online data supplement for more detail).

The PiB retention index as the standardized uptake value ratio (SUVR) for each region of interest (ROI) was calculated by dividing the regional mean value by the individual mean cerebellar uptake values. The automatic anatomic labeling algorithm [26] and a region combining method [27] were applied to set the ROIs to characterize PiB retention level in frontal, lateral parietal, posterior cingulate-precuneus (PC-PRC), and lateral temporal regions. Each participant was classified as Aβ-positive if the SUVR value was > 1.4 in at least one of the four ROIs or as Aβ-negative if the SUVR values of all four ROIs was ≤ 1.4 [27, 28]. For the ROI analyses, a voxel-number weighted average SUVR of a composite global ROI was calculated using the four ROIs (AD_PiB_-ROI).

### Cerebral glucose imaging

All subjects also underwent three-dimensional [^18^F] fluoro-2-deoxyglucose (FDG) PET taken using the same PET-MR scanner as the PiB-PET. The acquisition parameters were similar to the PiB-PET procedures (described in more detail in Additional file 1: Methods).

In terms of spatial normalization processes performed on FDG-PET data, basic preprocessing was the same as for PiB-PET described above. The spatially normalized FDG-PET images were smoothed with a 12-mm Gaussian filter. For FDG-PET images, intensity normalization was performed on spatially normalized images using pons as the reference region, and SUVRs were extracted for ROIs using the standard AAL 116 atlas. For the ROI analyses, a voxel-number weighted average SUVR of a composite ROI (AD_FDG_-ROI) including middle temporal gyrus, posterior cingulate cortex (PCC), and fusiform gyrus was calculated, which are the regions known to be sensitive to metabolic changes associated with AD [17].

### Statistical analysis

Analyses were performed with SPSS 23.0 and SPM12. The APOE genotypes were coded as APOE4 carrier (APOE4^+^) or noncarrier (APOE4^–^). Differences in clinical and ROI measures between the FH groups as well as the APOE4 groups were examined with independent sample *t* tests, a general linear model (GLM), and *χ*^*2*^ tests. Main effects and interaction were examined in the model, adjusting for age and gender effects. Post-hoc tests for an interaction effect were performed using Dunn-Sidak correction for multiple comparisons. In addition, the APOE4 carrier status was added as a covariate for main effects model of FH; likewise, the FH status was added to covariates for the main effects model of APOE4. Results were examined at *p* < 0.05.

To explore the interaction effects with more detailed regional information, the GLM was used to test for regional differences in parametric PiB and FDG SUVR images using SPM12. Results were initially examined at *p* < 0.005, uncorrected for multiple comparisons. A significant cluster was identified based on a cluster correction procedure available in the Analysis of Functional NeuroImage (3dClustSim, version built 4 November 2016), which performed 10,000 iterations of Monte Carlo simulations on anatomical mask datasets with 1,801,748 voxels. This method, derived from Gaussian Random Field Theory, protects against multiple comparisons [29]. The cluster size threshold to achieve correction for multiple comparisons at *p* < 0.05 was calculated to be *k* > 1062 voxels.

## Results

### Participant characteristics

Demographic characteristics are shown in Table 1. Of the 268 study participants, 51 (19%) had at least one first-degree family member with a history of late-onset AD. Each participant was identified into one of the four groups: FH^–^APOE4^–^, FH^+^APOE4^–^, FH^–^POE4^+^, or FH^+^APOE4^+^. There were no differences between the groups regarding age, gender distribution, education, or cognitive functioning. The total allele frequency of APOE4 in this cohort was 9%, which is consistent with a previous report on APOE polymorphism among Koreans [30]. There were two APOE4 homozygote carriers in the entire sample, both of whom were FH^+^. The proportion of Aβ-positive subjects was significantly higher in the FH^+^APOE4^+^ group than the other groups.

**Table 1:** Subject characteristics by family history and APOE4 groups

	FH^–^APOE4^–^	FH^+^APOE4^–^	FH^–^APOE4^+^	FH^+^APOE4^+^	*P*
*n*	180	38	37	13	
Age (years), mean (SD)	68.2 (8.2)	65.2 (9.5)	69.6 (9.4)	68.2 (9.5)	0.41
Gender, female/male (% female)	97/83 (53.9)	15/23 (39.5)	20/17 (54.1)	6/7 (46.2)	0.12
Education (years), mean (SD)	11.9 (4.9)	13.4 (4.1)	11.0 (4.1)	12.2 (4.4)	0.15
APOE4 dosage, *n* of ε4/ε4 (%)	0 (0)	0 (0)	0 (0)	2 (15)	
MMSE (raw score), mean (SD)	27.0 (2.4)	27.5 (2.4)	26.2 (2.9)	27.3 (2.5)	0.11
Neuropsychological test performance (*z* score, mean (SD))					
Immediate Verbal Memory Free Recall	0.88 (0.96)	0.86 (0.98)	0.60 (1.02)	0.81 (0.73)	0.46
Delayed Verbal Memory Free Recall	0.41 (0.87)	0.54 (0.89)	0.29 (0.90)	0.53 (0.62)	0.60
Delayed Verbal Memory Recognition	0.19 (0.78)	0.28 (0.65)	−0.003 (0.84)	0.27 (0.69)	0.40
Delayed Nonverbal Memory	0.33 (0.92)	0.49 (0.74)	0.05 (0.82)	0.45 (0.58)	0.14
Semantic Fluency	0.30 (1.12)	0.51 (1.18)	−0.09 (0.80)	0.75 (1.58)	0.05
Confrontational Naming	0.52 (0.89)	0.65 (0.63)	0.35 (0.87)	0.87 (0.58)	0.22
Constructional Praxis	−0.04 (0.93)	0.21 (0.66)	−0.08 (1.13)	−0.10 (1.04)	0.46
Stroop Color-Word	0.23 (1.04)	0.45 (0.91)	−0.001 (0.76)	0.27 (0.91)	0.27
Trail Making Test A	0.65 (1.93)	1.03 (0.46)	0.48 (1.91)	0.85 (0.50)	0.54
Trail Making Test B^a^	0.89 (1.04)	1.40 (0.85)	0.94 (1.14)	0.96 (0.73)	0.11
Digit Span Forward	0.36 (1.07)	0.52 (0.90)	−0.04 (0.98)	0.58 (0.92)	0.08
Digit Span Backward	0.12 (1.23)	0.52 (1.34)	−0.24 (0.86)	0.38 (1.81)	0.06
Amyloid-beta positive, *n* (%)	19 (11)	5 (13)	7 (19)	5 (38)*	0.03

*APOE4* apolipoprotein ε4 allele, *APOE4*^*+*^ APOE4 carrier, *APOE4*^*–*^ APOE4 noncarrier, *FH* parental or sibling (first-degree relative) family history of late-onset AD (age of onset ≥ 65 years), *FH*^*+*^ positive FH, *FH*^*–*^ negative FH, *MMSE* Mini-Mental State Examination, *SD* standard deviation

^a^35% of FH^–^APOE4^–^, 24% of FH^+^APOE4^–^, 59% of FH^–^APOE4^+^, and 38% of FH^+^APOE4^+^ did not complete the Trail Making Test B due to cognitive reasons, participation refusal, or illiteracy

*Significantly different from the other groups based on post-hoc analyses, multiple comparisons corrected (*P* = 0.0067). Homozygote APOE4 carriers were not among the five amyloid-beta-positive individuals

### Independent association of FH and APOE4 on cerebral amyloid deposition

**Fig. 1:**
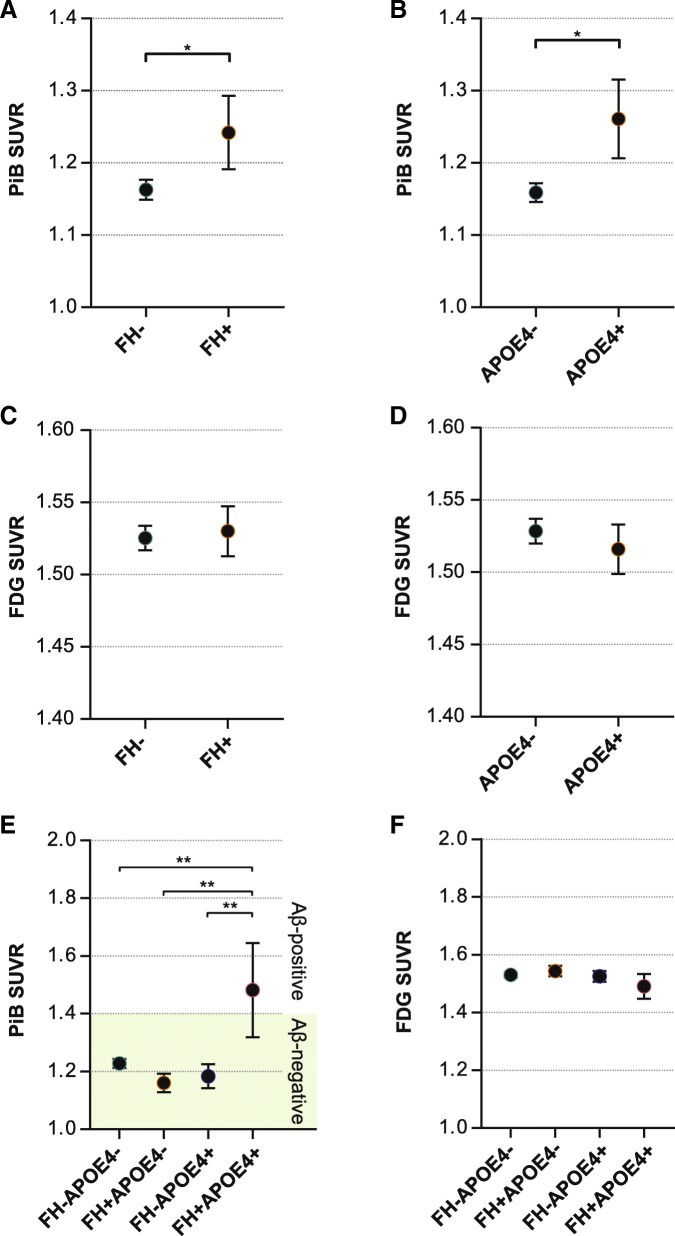
Mean PiB retention and FDG uptake SUVRs. **a** AD_PiB_-ROI between FH^–^ and FH^+^. **b** AD_PiB_-ROI between APOE4^–^ and APOE4^+^. **c** AD_FDG_-ROI between FH^–^ and FH^+^. **d** AD_FDG_-ROI between APOE^–^ and APOE^+^. **e** Interaction effect of FH and APOE on PiB retention. **f** Interaction effect of FH and APOE on FDG uptake. The green shaded area in **e** denotes below the PiB SUVR threshold of 1.4. Error bars indicate standard error. *P < 0.05; **significant difference in the post-hoc analyses adjusted for multiple comparisons using Dunn-Sidak correction (*P*_*B*_ < 0.0085). Aβ amyloid beta, APOE4 apolipoprotein ε4 allele, APOE4^+^ APOE4 carriers, APOE4^–^ APOE4 noncarriers, FDG [^18^F] fluoro-2-deoxyglucose, FH family history of Alzheimer’s disease, FH^+^ individuals with FH, FH^–^ individuals without FH, L left hemisphere, PiB [^11^C] Pittsburg compound B, R right hemisphere, SUVR standardized uptake value ratio

Significant differences between FH^–^ and FH^+^ were found for the AD_PiB_-ROI (Additional file 1: Table S3 (Model-A)) and remained the same when the effect was additionally adjusted for APOE4 status (Fig. 1a; Additional file 1: Table S3 (Model-B)).

Significant differences in Aβ deposition levels were found in the AD_PiB_-ROI between the APOE4^+^ and APOE4^–^ groups (Additional file 1: Table S3 (Model-C)). Significant differences remained the same when FH status was added as a covariate (Fig. 1b; Additional file 1: Table S3 (Model-D)). Overall, PiB retention was higher in FH^+^ compared with FH^–^ and in APOE4^+^ compared with APOE4^–^.

### Independent association of FH and APOE4 on cerebral glucose metabolism

There were no significant differences found in the AD_FDG_-ROI between the FH groups (Additional file 1: Table S3 (Model-A)) or the APOE4 groups (Additional file 1: Table S3 (Model-C)). Results remained the same when APOE4 status was added as a covariate (Fig. 1c; Additional file 1: Table S3 (Model-B)) or when FH status was added as a covariate (Fig. 1d; Additional file 1: Table S3 (Model-D)).

### FH-APOE4 interaction effects: ROI analyses

A significant FH-APOE4 interaction effect was found for the AD_PiB_-ROI (*F* = 11.51, *p* < 0.001, *R*^*2*^ = 0.112) in addition to significant main effects of FH and APOE4 (Additional file 1: Table S1); the FH^+^APOE4^+^ group showed significantly higher Aβ deposition compared with the other groups (Fig. 1e). However, there were no main or interaction effects on regional cerebral glucose metabolism (rCMglc) between FH and APOE4 (Fig. 1f; Additional file 1: Table S1). The interaction effects remained significant when two APOE4 homozygote individuals were excluded (Additional file 1: Table S6).

### FH-APOE4 interaction effects: voxel-wise analyses

**Fig. 2:**
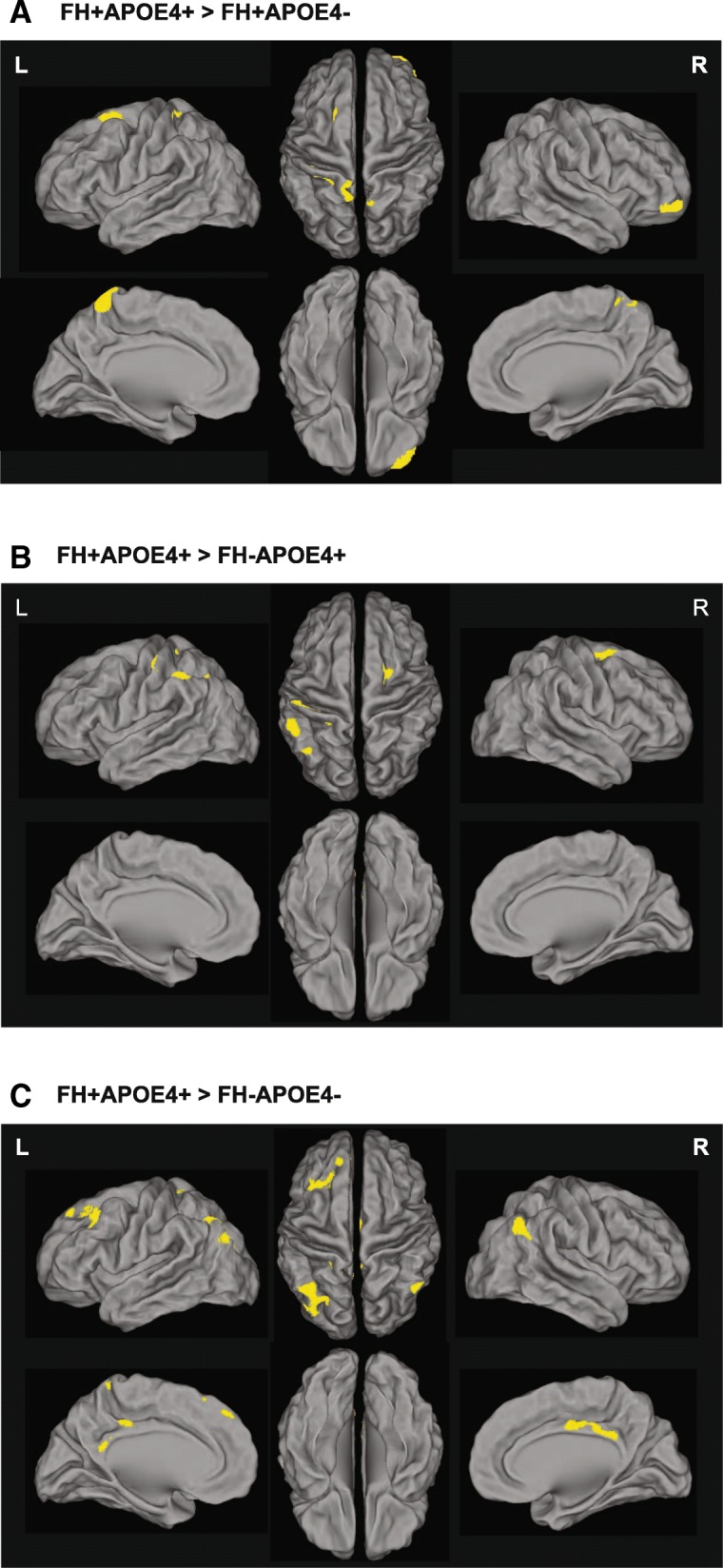
Regions showing significant differences in Aβ deposition from voxel-wise analyses. **a** FH^+^APOE4^+^ compared with FH^+^APOE4^–^. **b** FH^+^APOE4^+^ compared with FH^–^APOE4^+^. **c** FH^+^APOE4^+^ compared with FH^–^APOE4^–^. A anterior, APOE4 apolipoprotein ε4 allele, APOE4^+^ APOE4 carriers, APOE4^–^ APOE4 noncarriers, FH family history of Alzheimer’s disease, FH^+^ individuals with FH, FH^–^ individuals without FH, L left hemisphere, P posterior, R right hemisphere

Voxel-wise analyses were conducted between the FH^+^APOE4^+^ group and the other groups to further explore detailed brain regions showing the interaction effects on Aβ deposition and rCMglc. Compared with FH^+^APOE4^–^, FH^+^APOE4^+^ showed increased Aβ deposition in the left postcentral gyrus, left superior frontal gyrus, and left precuneus (Fig. 2a); compared with FH^–^APOE4^+^, FH^+^APOE4^+^ showed increased Aβ deposition in the left postcentral and supramarginal gyri and left superior frontal gyrus (Fig. 2b); and compared with FH^–^APOE4^–^, FH^+^APOE4^+^ showed increased Aβ deposition in the left middle frontal and temporal gyri, right inferior parietal lobule, left postcentral gyrus, and left posterior cingulate gyrus (Fig. 2c). There were no regions in any of the comparisons where FH^+^APOE4^+^ showed lower Aβ deposition levels (Additional file 1: Table S4).

**Fig. 3:**
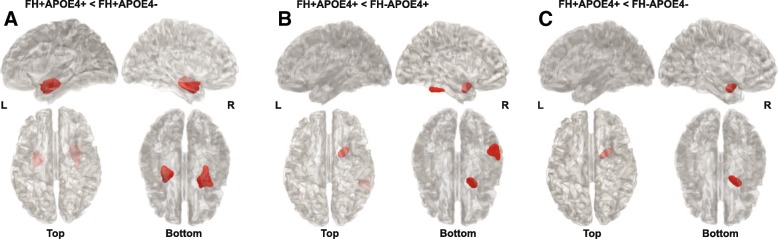
Regions showing significant difference in cerebral glucose metabolism from voxel-wise analyses. **a** FH^+^APOE4^+^ compared with FH^+^APOE4^–^. **b** FH^+^APOE4^+^ compared with FH^–^APOE4^+^. **c** FH^+^APOE4^+^ compared with FH^–^APOE4^–^. APOE4 apolipoprotein ε4 allele, APOE4^+^ APOE4 carriers, APOE4^–^ APOE4 noncarriers, FH family history of Alzheimer’s disease, FH^+^ individuals with FH, FH^–^ individuals without FH, L left hemisphere, R right hemisphere

Although there were no significant interaction effects of FH and APOE4 on rCMglc found from the ROI analyses, voxel-wise analyses were conducted for explorative purposes since subtle changes in CN adults may not have been captured by the AD_FDG_-ROI. Compared with FH^+^APOE4^–^, FH^+^APOE4^+^ showed decreased rCMglc in the right entorhinal area and left hippocampus (Fig. 3a); compared with FH^–^APOE4^+^, FH^+^APOE4^+^ showed decreased rCMglc in the right entorhinal area and inferior temporal gyrus (Fig. 3b); and compared with FH^–^APOE4^–^, FH^+^APOE4^+^ showed decreased rCMglc in the right entorhinal area (Fig. 3c). There were no regions with significant differences in any of the comparisons where FH^+^APOE4^+^ showed hypermetabolism compared with the other groups (Additional file 1: Table S4).

## Discussion

The present study examined whether APOE4 and FH have synergistic interaction effects on cerebral Aβ deposition and glucose metabolism in healthy middle-aged and older adults. The most notable finding was a significant increase in Aβ deposition if an individual was an APOE4 carrier with a positive family history for AD. While the synergistic interaction effect of FH and APOE4 on cerebral glucose metabolism was not found in the ROI analyses, voxel-wise analyses revealed that the synergistic interaction effect on glucose metabolism was in fact present in the temporal regions, including the hippocampi. To the best of our knowledge, there is no previous study examining the interaction effects of APOE4 and FH on both Aβ deposition and rCMglc.

A body of literature devoted to investigating the genetic risks of AD has largely focused on either APOE4 or FH. The current findings on the interaction effects of FH and APOE4 on Aβ deposition and rCMglc further delineate the nature of the relationship between the two risk factors on signature AD brain changes. As can be inferred from the interaction effects, differences in the proportions of FH^+^ individuals included in the study samples may account, at least in part, for varied magnitudes of the effects of APOE4 reported in the literature. Conceivably, the commonly reported effects of APOE4 on AD brain biomarkers in the literature may actually be the effects seen in FH^+^APOE4^+^ compared with other groups. A similar argument can be made regarding the investigations of the effects of FH on AD brain biomarkers since the effects of FH were only seen in APOE4 carriers. Given the significant synergistic interaction effect, a simple application of statistical adjustment to achieve independency of the effects of either one of the risk factors may be misrepresenting.

A question arises as to the role played by a family history of AD in APOE4 carriers. The results from the current study strongly suggest that the APOE4 carriers with a family history of AD are more susceptible to alterations of the signature AD brain biomarkers before the onset of any cognitive symptoms. A positive family history status may represent other susceptibility genes that are either coexpressed or interacting with APOE4. It has been posited that gene-gene interactions account for much of the unexplained variances in AD status [31] and that these interactions are widespread and common [32]. For example, clusterin (CLU; involved in AD pathogenesis directly by influencing Aβ aggregation and clearance [33, 34]) is found to interact with APOE4 [35, 36]. Bridging integrator 1, whose role is implicated in tauopathy [37], is thought to interact with the CLU [38]. In addition to the known genetic risks for AD possibly interacting with each other, there is a possibility that FH^+^ is capturing additional genetics that are considered unrelated to AD but interact with the AD genetic risks. Delineation of the role of genetics in the diagnosis and risk prediction in late-onset AD is complicated since up to 75% of APOE4 carriers do not develop AD and up to 50% of individuals with AD are APOE4 noncarriers [39, 40]. A twin study that examined genetic and environmental influences on AD reported that additive genetic influences explain 79% of the variance in the AD phenotype as opposed to 21% explained by nonshared environmental influences; when shared environmental influences were added to the analysis model, however, the variance in the AD phenotype explained by additive genetic influences changed to 58%, and 42% of the variance was explained by environmental influences (both shared (19%) and nonshared (23%)) [12]. Furthermore, a more recent study using the Alzheimer’s Disease Genetics Consortium data and Genome-wide Complex Trait Analysis method reported that 53.24% of phenotypic variance was explained by genetics [41]. Given that environmental factors likely can explain the variance unaccounted for by genetic risks, involvement of a family’s shared environments (captured by FH^+^) needs to be considered carefully as they may interact with APOE4 and other genetic risks. Shared environmental factors, which are seldom reported in association with Aβ, include a family’s socioeconomic status, place of living (e.g., urban versus rural), lifestyle or dietary habits, parents’ educational attainment and intellectual environment, and exposure to pollution [42–46]. Cognitive activity during the early life stage, for example, has been found to be associated with reduced neurodegeneration in AD signature regions in later life [47]. Although the abovementioned environmental factors may not individually show strong effects on AD, a combination of the environmental factors with APOE4 or other genetic risks may exhibit meaningful influences on AD neuropathology.

While independent main effects of APOE4 and FH were observed on Aβ deposition, there were no differences in cerebral glucose metabolism between the FH groups or the APOE4 groups in the brain regions typically affected in clinical AD patients in the current sample of cognitively normal elderly whose degree of degeneration is not yet progressed enough to be detected. In line with the findings by Lowe et al. [5] who showed that most of the APOE4-related differences in hypometabolism are mediated by amyloid accumulation, hypometabolism associated with APOE4 or FH in the AD signature regions in the current sample was only observed in individuals with high Aβ deposition (Additional file 1: Figure S1, Table S5), suggesting that the effects of APOE4 or FH on cerebral glucose metabolism only begin to show when the disease is progressed. Moreover, the hypometabolic pattern observed, particularly in the medial temporal regions, in association with FH^+^ and APOE4^+^ status (Fig. 3) is noteworthy given that these regions are where phosphorylated tau (p-tau) pathology is first to develop prior to accumulation of neurofibrillary tangles [8]. Taken together, the current cross-sectional observation in the CN likely reflects early observation of abnormal Aβ biomarkers prior to the onset of changes in glucose metabolism in the AD signature regions, which supports the amyloid pathological cascade model [48].

A strength of the current study is that the study participants were recruited from the community and that the sample includes a large number of APOE4^+^ and FH^+^ individuals despite relatively lower proportions (18% and 19%, respectively) compared with previous studies (24–43% and up to 66%, respectively) [5, 6, 8, 9, 16, 18]. The studies based on the Alzheimer’s Disease Neuroimaging Initiative (ADNI) data, for instance, frequently recruited from family members of AD patients such that the samples included a large proportion of FH^+^ individuals, and these samples included a large proportion of Aβ-positive CN individuals (e.g., 47%) [1, 5, 49], which is considerably higher than the current sample (13%). The current findings on the independent effects of APOE4 and FH on Aβ deposition and glucose metabolism are therefore relatively robust from differences in the proportions of APOE4^+^ or the number of FH^+^ individuals included in the total sample.

Similar to previous studies on FH, steps were taken to ensure that AD diagnosis in the subjects’ parents was accurate, such as including only the subjects whose parent’s AD diagnosis was made by a clinician according to the established criteria. Also, in a few cases where parents were deceased before formal diagnosis was available in clinics (i.e., parents of our old-old participants), a thorough interview was conducted with family members by a psychiatrist with expertise in dementia research to sufficiently address the inclusion/exclusion criteria of the AD diagnosis. Nonetheless, our cohort may have included a few participants whose parents did not have AD but instead had other types of dementia since the information obtained regarding those parents without a formal diagnosis is subject to recall bias. In the future, an investigation into possible protective effects of the APOE ε2 allele will allow further understanding of its role in the interaction effect of APOE and FH.

## Conclusions

The current study was the first attempt to elucidate the interaction effects between FH and APOE4 on cerebral Aβ deposition and glucose metabolism in cognitively healthy adults. The strong synergistic effects of APOE4 and FH on brain Aβ deposition and hypometabolism that were found indicate possible gene-to-gene or gene-to-environment interactions that are important for the pathogenesis of AD. Such a synergistic effect also indicates that individuals with both FH and APOE4 are the population that needs more attention with regard to preventive interventions.

## Additional file

Additional file 1: Supplementary material including additional methods, results, and author list for the KBASE group. (DOCX 1677 kb)

## Abbreviations

Aβ: Amyloid-beta
AD: Alzheimer’s disease
AD_FDG_-ROI: Composite global region of interest of FDG uptake
AD_PiB_-ROI: Composite global region of interest of PiB retention
APOE4: Apolipoprotein ε4 allele
CERAD-K: Consortium to Establish a Registry for Alzheimer’s Disease, Korean version
CLU: Clusterin
CN: Cognitively normal
FDG: [^18^F] fluoro-2-deoxyglucose
FH: Family history of Alzheimer’s disease
GLM: General linear model
KBASE: Korean Brain Aging Study for the Early Diagnosis and Prediction of AD
MNI: Montreal Neurological Institute
MRI: Magnetic resonance imaging
PCC: Posterior cingulate cortex
PC-PRC: Posterior cingulate-precuneus
PET: Positron emission tomography
PiB: [^11^C] Pittsburg compound B
rCMglc: Regional cerebral glucose metabolism
ROI: Region of interest
SUVR: Standardized uptake value ratio
