# Supplementary material for: Synergistic interaction between APOE and family history of Alzheimer’s disease on cerebral amyloid deposition and glucose metabolism
Source: Alzheimers Res Ther. 2018 Aug 23;10:84. doi: 10.1186/s13195-018-0411-x (PMC6106945; doi:10.1186/s13195-018-0411-x)
Supplement: Supplementary file 1 — Supplementary material including additional methods, results, and author list for the KBASE group. (DOCX 1677 kb) [file 13195_2018_411_MOESM1_ESM.docx]

**Synergistic interaction between APOE and family history of Alzheimer’s disease on cerebral amyloid deposition and glucose metabolism**

***Additional file***

**SI Methods**

**Amyloid beta imaging acquisition**

PiB was synthesized on site. For PiB-PET imaging, participants fasted for at least 6 hours prior to receiving intravenous administration of the radioligands (555 MBq (range 450~610 MBq) of PiB). After the injections, subjects rested for 40 minutes in a dimly lit waiting room prior to getting scanned. The PET data collected in list mode were processed for routine corrections such as uniformity, UTE-based attenuation, and decay corrections, and reconstructed into a 256 x 256 image matrix using iterative methods (6 iterations with 21 subsets). T1-weighted (repetition time = 1670 ms, echo time = 1.89 ms, field of view = 250 mm, 256 X 256 matrix with 1.0 mm slice thickness) were acquired in the sagittal orientation.

**Cerebellar Grey Matter Spatial Normalization**

Additional steps of preprocessing were run for PiB-PET data to obtain improved spatial normalization of cerebellar grey matter, which is used as the reference region for intensity normalization. Of the twenty-eight anatomical structural regions in the cerebellar atlas, the cerebellar lobular regions except for vermis were included to extract the mean cerebellar uptake values to be used to generate intensity normalized PiB-PET images. In order to eliminate inclusion of nonspecific binding in the white matter, the transformation parameters of PiB-PET obtained from the abovementioned spatial normalization steps were inversed using the Individual Brain Atlases using Statistical Parametric Mapping software and used to bring the standard automatic anatomic labeling (AAL) 116 atlas to individual space of each subject (a resampling voxel size = 1 x 0.98 x 0.98 mm), and non-gray matter portion of the atlas were individually masked using cerebral gray matter segment image of each subject.

**Cerebral glucose imaging acquisition**

Participants fasted for at least 6 hours prior to receiving intravenous administration of the radioligands (0.1 mCi/Kg of FDG). After the injections, subjects rested for 40 minutes in a dimly lit waiting room prior to getting scanned. The PET data collected in list mode were processed for routine corrections such as uniformity, UTE-based attenuation, and decay corrections, and reconstructed into a 256 x 256 image matrix using iterative methods (6 iterations with 21 subsets). T1-weighted (repetition time = 1670 ms, echo time = 1.89 ms, field of view = 250 mm, 256 X 256 matrix with 1.0 mm slice thickness) were acquired in the sagittal orientation.

**Supplementary Figure S1. Interaction Effects on Glucose Metabolism by Amyloid Positivity**


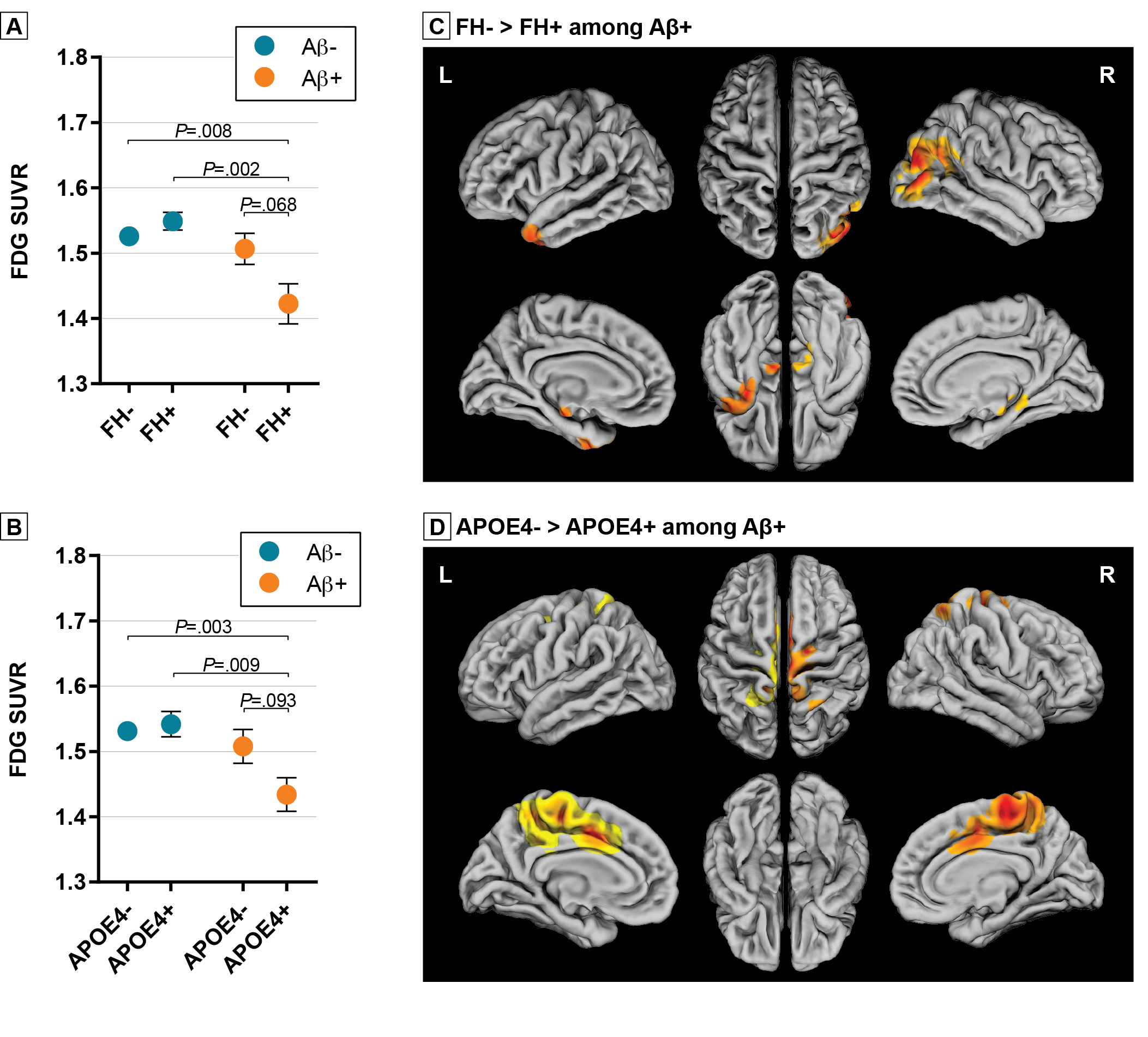


Interaction effects between FH and Aβ positivity (A) and between APOE4 and Aβ positivity (B) on FDG uptake. Voxel-wise comparisons between FH- and FH+ among Aβ+ individuals (C) and between APOE4- and APOE4+ among Aβ+ individuals (D).

Abbreviations: FH, family history of Alzheimer’s disease; APOE4, apolipoprotein ε4 allele; Aβ, amyloid beta; Aβ+, amyloid beta positive; Aβ-, amyloid beta negative; APOE4+, apolipoprotein ε4 allele carrier; APOE4+, apolipoprotein ε4 allele non-carrier; L, left hemisphere; R, right hemisphere; FDG, [^18^F] fluoro-2-deoxyglucose; SUVR, standardized uptake value ratio.

**Supplemental Table S1. AD-signature PET ROI measures by family history groups and APOE4 status**

| Variables |  |  | Model-A | | Model-B | |  |  | |  | Model-C | | Model-D | |
| --- | --- | --- | --- | --- | --- | --- | --- | --- | --- | --- | --- | --- | --- | --- |
|  | FH- | FH+ | *F* | *P* | *F* | *P* |  | APOE4- | APOE4+ | | *F* | *P* | *F* | *P* |
| AD_PiB_-ROI | 1.16 (0.20) | 1.24 (0.36) | 6.69 | .01* | 5.65 | .02* |  | 1.16 (0.19) | 1.26 (0.38) | | 6.29 | .01* | 5.25 | .02* |
| AD_FDG_-ROI | 1.53 (0.13) | 1.53 (0.12) | 0.03 | .86 | 0.02 | .89 |  | 1.53 (0.13) | 1.52 (0.12) | | 0.19 | .66 | 0.18 | .68 |

*Note.* Values are mean(SD).

Abbreviations: Aβ, beta-amyloid; AD, Alzheimer’s disease; ROI, region of interest; APOE4, the apolipoprotein ε4 allele; APOE4+, APOE4 carriers; APOE4-, APOE4 non-carriers; FH, parental or sibling (1^st^ degree relative) family history of late onset AD (age of onset ≥ 65); FH-, negative FH; FH+, positive FH; AD_PiB_-ROI, global cortical ROI PiB SUVR; AD_FDG_-ROI, composite ROI FDG SUVR; Model A, adjusted for age and gender; Model B, adjusted for age, gender, and APOE4; Model C, adjusted for age and gender; Model D, adjusted for age, gender, and FH.

**P* < .05

**Supplemental Table S2. The influence of FH, APOE4 and their interaction on PiB retention and FDG uptake**

| **Variables** | ***F*-test** | ***p*-value** | **Effect Size**  **(partial eta squared)** | **Observed Power** |
| --- | --- | --- | --- | --- |
| AD_PiB_-ROI |  |  |  |  |
| Corrected Model | 7.77 | .000 | .129 | .999 |
| Intercept | 52.89 | .000 | .168 | 1.000 |
| Age | 14.38 | .000 | .052 | .965 |
| Sex | 0.00 | .983 | .000 | .050 |
| APOE4 Status | 14.16 | .000 | .051 | .963 |
| FH Status | 14.74 | .000 | .053 | .969 |
| FH x APOE4 | 11.51 | .001 | .042 | .922 |
|  |  |  |  |  |
|  |  |  |  |  |
| AD_FDG_-ROI |  |  |  |  |
| Corrected Model | 2.18 | .057 | .040 | .710 |
| Intercept | 766.56 | .000 | .745 | 1.000 |
| Age | 8.79 | .003 | .032 | .840 |
| Sex | 0.55 | .460 | .002 | .114 |
| APOE4 Status | 0.81 | .369 | .003 | .146 |
| FH Status | 0.43 | .514 | .002 | .100 |
| FH x APOE4 | 1.08 | .299 | .004 | .179 |

*Note.* AD, Alzheimer’s disease; ROI, region of interest; APOE4, the apolipoprotein ε4 allele; APOE4+, APOE4 carriers; APOE4-, APOE4 non-carriers; FH, parental or sibling (1^st^ degree relative) family history of late onset AD (age of onset ≥ 65); AD_PiB_-ROI, global cortical ROI PiB SUVR; AD_FDG_-ROI, composite ROI FDG SUVR

**Supplemental Table S3. Post hoc tests of the interaction effects of FH and APOE4 on PiB retention**

| Variables | FH x APOE4 | | | *Post hoc* |
| --- | --- | --- | --- | --- |
|  | *F* | *R^2^* | *P* |  |
| AD_PiB_-ROI | 11.51 | .129 | .001 | FH+APOE4+ > FH+APOE4-*; FH+APOE4+ > FH-APOE4+*; FH+APOE4+ > FH-APOE4-* |

Abbreviations: APOE4, the apolipoprotein ε4 allele; APOE4+, APOE4 carriers; APOE4-, APOE4 non-carriers; FH, parental or sibling (1^st^ degree relative) family history of late onset AD (age of onset ≥ 65); FH-, negative FH; FH+, positive FH; AD_PiB_-ROI, global cortical ROI PiB SUVR

*Note.* Adjusted for age and gender.

*Significant *post hoc* after Dunn-Sidak correction (*P_B_* < .00071)

**Supplemental Table S4. Interaction Effects of FH and APOE4 on PiB Retention and FDG Uptake without APOE4 Homozygotes**

| Variables | FH x APOE4 | | | *Post hoc* |
| --- | --- | --- | --- | --- |
|  | *F* | *R^2^* | *P* |  |
| AD_PiB_-ROI | 8.97 | .138 | .000011 | FH+APOE4+ > FH+APOE4-*; FH+APOE4+ > FH-APOE4+*; FH+APOE4+ > FH-APOE4-* |
| AD_FDG_-ROI | 0.66 | .05 | .58 |  |

Abbreviations: APOE4, the apolipoprotein ε4 allele; APOE4+, APOE4 carriers; APOE4-, APOE4 non-carriers; FH, parental or sibling (1^st^ degree relative) family history of late onset AD (age of onset ≥ 65); FH-, negative FH; FH+, positive FH; AD_PiB_-ROI, global cortical ROI PiB SUVR; AD_FDG_-ROI, composite ROI FDG SUVR.

*Note.* Adjusted for age and gender.

*Significant *post hoc* after Dunn-Sidak correction (*P_B_* < .00071)

**Supplemental Table S5. Brain regions showing significant difference in amyloid beta deposition in the FH+APOE4+ group compared to the other groups**

|  | | Coordinates* | | | Cluster Extent | *T*^§^ | Brodmann area |
| --- | --- | --- | --- | --- | --- | --- | --- |
|  |  | x | y | z |  |  |  |
| Aβ deposition |  |  |  |  |  |  |  |
| FH+APOE4+ > FH+APOE4- | Postcentral gyrus, left | -35 | -31 | 62 | 3843 | 3.31 | 4 |
|  | Superior frontal gyrus, left | -12 | -45 | 69 | 1338 | 3.18 | 4/5 |
|  | Precuneus, left | -9 | -52 | 65 | 1594 | 2.91 | 7 |
| FH+APOE4+ < FH+APOE4- | No regions |  |  |  |  |  |  |
| FH+APOE4+ > FH-APOE4+ | Postcentral/supramarginal gyrus, left | -36 | -29 | 62 | 5143 | 3.69 | 4/2/40 |
|  | Superior frontal gyrus, right | 22 | 5 | 64 | 1133 | 3.07 | 6 |
| FH+APOE4+ < FH-APOE4+ | No regions |  |  |  |  |  |  |
| FH+APOE4+ > FH-APOE4- | Middle frontal gyrus, left | -38 | 22 | 47 | 2927 | 3.06 | 8 |
|  | Middle temporal gyrus, left | -38 | -74 | 28 | 4551 | 3.05 | 39 |
|  | Inferior parietal lobule, right | 45 | -60 | 36 | 1724 | 2.93 | 40 |
|  | Postcentral gyrus, left | -14 | -42 | 66 | 1138 | 2.94 | 3 |
|  | Posterior cingulate gyrus, left | -7 | -35 | 41 | 4508 | 2.83 | 31 |
| FH+APOE4+ < FH-APOE4- | No regions |  |  |  |  |  |  |
| Cerebral glucose metabolism |  |  |  |  |  |  |  |
| FH+APOE4+ < FH+APOE4- | Entorhinal area, right | 26 | 3 | -22 | 4778 | 3.56 | 28/34 |
|  | Hippocampus, left | -28 | -11 | -19 | 3453 | 3.40 |  |
| FH+APOE4+ > FH+APOE4- | No regions |  |  |  |  |  |  |
| FH+APOE4+ < FH-APOE4+ | Entorhinal area, right | 22 | 5 | -22 | 1930 | 3.62 | 34 |
|  | Inferior temporal gyrus, right | 53 | -45 | -28 | 1670 | 3.24 | 37 |
| FH+APOE4+ > FH-APOE4+ | No regions |  |  |  |  |  |  |
| FH+APOE4+ < FH-APOE4- | Entorhinal area, right | 18 | 0 | -24 | 1674 | 3.22 | 34 |
| FH+APOE4+ > FH-APOE4- | No regions |  |  |  |  |  |  |

*Coordinates from MNI space.

^§^*T* values at the peak of maximum significance at *p*<0.005, uncorrected. Results are significant at level *P* < .005, *k* > 1062 voxels based on cluster correction procedure, controlling for age and gender.

Abbreviations: Aβ, beta-amyloid; APOE4, the apolipoprotein ε4 allele; APOE4+, APOE4 carriers; APOE4-, APOE4 non-carriers; FH, parental or sibling (1st degree relative) family history of late onset AD (age of onset ≥ 65); FH-, negative FH; FH+, positive FH.

**Supplemental Table S6. Brain regions showing significant difference in cerebral glucose metabolism**

|  | | Coordinates* | | | Cluster Extent | *T*^§^ | Brodmann area |
| --- | --- | --- | --- | --- | --- | --- | --- |
|  |  | x | y | z |  |  |  |
| Among Aβ positive |  |  |  |  |  |  |  |
| APOE4- > APOE4+ | Middle cingulate gyrus, left | -9 | 1 | 40 | 47703^♯^ | 4.46 | 24 |
|  | Medial frontal gyrus, left | -11 | -22 | 55 |  | 4.01 | 6 |
|  | Medial frontal gyrus, right | 15 | -18 | 62 |  | 3.97 | 6 |
|  | Superior parietal lobule, right | 28 | -61 | 61 | 1227 | 3.4 | 7 |
| APOE4- < APOE4+ | No regions |  |  |  |  |  |  |
| FH- > FH+ | Middle occipital gyrus, right | 46 | -85 | 12 | 13723 | 4.11 | 19 |
|  | Inferior occipital gyrus, right | 44 | -89 | 4 |  | 4.03 | 19 |
|  | Cerebellum, anterior lobe, right | 3 | -32 | -24 | 3846 | 3.93 |  |
|  | Midbrain | -4 | -23 | -21 |  | 3.74 |  |
|  | Temporal pole, left | -49 | 15 | -36 | 2406 | 3.89 | 38 |
|  | Inferior temporal gyrus, left | -29 | 2 | -47 |  | 3.63 | 20 |
| FH- < FH+ | No regions |  |  |  |  |  |  |
| Among Aβ negative |  |  |  |  |  |  |  |
| APOE4- > APOE4+ | No regions |  |  |  |  |  |  |
| APOE4- < APOE4+ | No regions |  |  |  |  |  |  |
| FH- > FH+ | No regions |  |  |  |  |  |  |
| FH- < FH+ | No regions |  |  |  |  |  |  |

*Coordinates from MNI space.

^§^*T* values at the peak of maximum significance at *P* < .005, uncorrected. Results are significant at level *P* < .005, *k* > 1062 voxels based on cluster correction procedure, controlling for age and gender.

^♯^Cluster FWE corrected significance (*P* = .003)

Abbreviations: Aβ, beta-amyloid; APOE4, the apolipoprotein ε4 allele; APOE4+, APOE4 carriers; APOE4-, APOE4 non-carriers; FH, parental or sibling (1st degree relative) family history of late onset AD (age of onset ≥ 65); FH-, negative FH; FH+, positive FH.

**Coinvestigators**

**KBASE Research Group**

Dong Young Lee, MD, PhD (Seoul National University, Principal Investigator); Min Soo Byun, MD, PhD (Seoul National University, Core PI Clinical & Executive); Dahyun Yi, PhD (Seoul National University, Core PI Neuropsychology); Yu Kyeong Kim, MD, PhD (SMG-SNU Boramae Medical Center, Core PI PET); Chul-Ho Sohn, MD, PhD (Seoul National University, Core PI MRI); Inhee Mook-Jung, PhD (Seoul National University, Core PI Biomarker); Murim Choi, PhD (Seoul National University, Core PI Genetics); Yu Jin Lee, MD, PhD (Seoul National University, Core PI Sleep), Seokyung Hahn, PhD (Seoul National University, Core PI Biostatistics); Hyun Jung Kim, MD (Changsan Convalescent Hospital, co-investigator); Mun Young Chang, MD (Chung-Ang University College of Medicine, co-investigator); Seung Hoon Lee, MD (Daerim St. Mary's Hospital, co-investigator); Jee Wook Kim, MD, PhD (Hallym University Dongtan Sacred Heart Hospital, co-investigator); Jong-Min Lee, PhD (Hanyang University, co-investigator); Dong Woo Lee, MD, PhD (Inje University Snaggye Paik Hospital, co-investigator); Bo Kyung Sohn, MD (Inje University Snaggye Paik Hospital, co-investigator); Seok Woo Moon, MD, PhD (Konkuk University Chungju Hospital, co-investigator); Man Ho Choi, PhD (Korea Institute of Science and Technology, co-investigator); Sang-Won Lee, PhD (Korea University, co-investigator); Hyewon Baek, MD (Kyunggi Provincial Hospital for the Elderly, co-investigator); Na Young Han, MD (National Research Center for Dementia, co-investigator); Jong-Won Kim, MD, PhD (Samsung Medical Center, co-investigator); Seung-Ho Ryu, MD, PhD (School of Medicine Konkuk University, co-investigator); Shin Gyeom Kim, MD, PhD (Soonchunhyang University Hospital Bucheon, co-investigator); Sun-Ho Han, PhD (Seoul National University, co-investigator); Jae Sung Lee, PhD (Seoul National University, co-investigator); Yun-Sang Lee, PhD (Seoul National University, co-investigator); Jong Inn Woo, MD, PhD (Seoul National University, co-investigator); Sang Eun Kim, MD, PhD (Seoul National University Bundang Hospital, co-investigator); Byung Chul Lee, PhD (Seoul National University Bundang Hospital, co-investigator); Gi Jeong Cheon, MD, PhD (Seoul National University Hospital, co-investigator); Koung Mi Kang, MD (Seoul National University Hospital, co-investigator); Jee-Eun Park, MD, PhD; (Seoul National University Hospital, co-investigator); Hyeong Gon Yu, MD, PhD (Seoul National University Hospital, co-investigator); Jun-Young Lee, MD, PhD (SMG-SNU Boramae Medical Center, co-investigator); Hyo Jung Choi, MD (SMG-SNU Boramae Medical Center, co-investigator); Young Min Choe, MD (University of Ulsan College of Medicine, Ulsan University Hospital, co-investigator); Woonhyung Ghim, MD (Seoul National University Hospital, research fellow); So Yeon Jeon, MD (Seoul National University Hospital, research fellow); Woo Jin Kim, MD, PhD (Seoul National University Hospital, research fellow); Kang Ko, MD (Seoul National University Hospital, research fellow); Jun Ho Lee, MD (Seoul National University Hospital, research fellow); Kyoungjin Chu (Seoul National University Hospital, psychologist); Younghwa Lee, BA (Seoul National University Hospital, psychologist); Donghwi Hwang, BS (Seoul National University, image analyst); Seugn Kwan Kang, BS (Seoul National University, image analyst); Seong A Shin, MS (Seoul National University, image analyst); Jeong Yeon Hwang, MD (Seoul National University, data analyst); Jong-Chan Park, BS (Seoul National University, data analyst); Jong-Ho Park, MS (Samsung Medical Center, genetic data analyst); Jieun Seo, BS (Seoul National University, genetic data analyst); Mi Ae Han, RN (Seoul National University Hospital, research coordinator); Eun A Jo, RN (Seoul National University Hospital, research coordinator); Gi Jung Jung, RN, MS (Seoul National University Hospital, research coordinator); Jin Hee Keum, RN (Seoul National University Hospital, research coordinator); Mi Sun Kim, RN (SMG-SNU Boramae Medical Center, research coordinator); Min Jeong Kim, RN (Seoul National University Hospital, research coordinator); Han Na Lee, RN (Seoul National University Hospital, research coordinator); Bo Eun Park, RN (Seoul National University Hospital, research coordinator); Ji Sun Shin, RN (Seoul National University Hospital, research coordinator); Yun Jung Hwang, MS (Seoul National University Hospital, researcher); Joon Hyung Jung, MD (Seoul National University Hospital, researcher); Kiyoung Sung, MD (Seoul National University Hospital, researcher); Eun Hye Kim, BS (Seoul National University, research assistant); Han Byul Choi, BA (National Research Center for Dementia, administrative staff)
